# A single-pass type I membrane protein, mannose-specific L-type lectin, potentially involved in the adhesion and invasion of *Cryptosporidium parvum*[Fn FN1]

**DOI:** 10.1051/parasite/2024051

**Published:** 2024-08-29

**Authors:** Xiaotian Zhang, Songying Sun, Wenchao Zhao, Luyang Wang, Guanda Liang, Yuexin Wang, Baiyi Cai, Longxian Zhang, Xiaoying Li, Sumei Zhang

**Affiliations:** 1 College of Veterinary Medicine, Henan Agricultural University Zhengzhou 450046 Henan PR China; 2 International Joint Research Laboratory for Zoonotic Diseases of Henan Zhengzhou 450046 Henan PR China; 3 Key Laboratory of Quality and Safety Control of Poultry Products, Ministry of Agriculture and Rural Affairs Zhengzhou 450046 Henan PR China; 4 Department of Medicine, University of Alabama at Birmingham Birmingham AL 35249 USA

**Keywords:** *Cryptosporidium parvum*, L-type lectin, Carbohydrate-binding protein, Adhesion, Invasion

## Abstract

*Cryptosporidium* is a globally distributed zoonotic protozoan parasite that can cause severe diarrhea in humans and animals. L-type lectins are carbohydrate-binding proteins involved in multiple pathways in animals and plants, including protein transportation, secretion, innate immunity, and the unfolded protein response signaling pathway. However, the biological function of the L-type lectins remains unknown in *Cryptosporidium parvum*. Here, we preliminarily characterized an L-type lectin in *C. parvum* (CpLTL) that contains a lectin-leg-like domain. Immunofluorescence assay confirmed that CpLTL is located on the wall of oocysts, the surface of the mid-anterior region of the sporozoite and the cytoplasm of merozoites. The involvement of CpLTL in parasite invasion is partly supported by experiments showing that an anti-CpLTL antibody could partially block the invasion of *C. parvum* sporozoites into host cells. Moreover, the recombinant CpLTL showed binding ability with mannose and the surface of host cells, and competitively inhibited the invasion of *C. parvum*. Two host cell proteins were identified by proteomics which should be prioritized for future validation of CpLTL-binding. Our data indicated that CpLTL is potentially involved in the adhesion and invasion of *C. parvum*.

## Abstract (fr)

*Cryptosporidium* est un parasite protozoaire zoonotique répandu dans le monde entier qui peut provoquer de graves diarrhées chez les humains et les animaux. Les lectines de type L sont des protéines liant les glucides impliquées dans de multiples voies chez les animaux et les plantes, notamment le transport des protéines, la sécrétion, l’immunité innée et la voie de signalisation de la réponse protéique dépliée. Cependant, la fonction biologique des lectines de type L reste inconnue chez *Cryptosporidium parvum*. Ici, nous avons caractérisé de manière préliminaire une lectine de type L chez *C. parvum* (CpLTL) qui contient un domaine de type jambe de lectine. Le test d’immunofluorescence a confirmé que CpLTL est localisée sur la paroi des oocystes, la surface de la région médio-antérieure du sporozoïte et le cytoplasme des mérozoïtes. L’implication de CpLTL dans l’invasion parasitaire est en partie étayée par des expériences montrant qu’un anticorps anti-CpLTL peut bloquer partiellement l’invasion des sporozoïtes de *C. parvum* dans les cellules hôtes. De plus, la CpLTL recombinante a montré une capacité de liaison avec le mannose et la surface des cellules hôtes et a inhibé de manière compétitive l’invasion de *C. parvum*. Deux protéines de cellules hôtes ont été identifiées par protéomique et devraient être prioritaires pour la validation future de la liaison avec CpLTL. Nos données indiquent que CpLTL est potentiellement impliquée dans l’adhésion et l’invasion de *C. parvum*.

## Introduction

*Cryptosporidium* spp. are zoonotic pathogens that cause severe diarrhea in humans and animals, and pose a great threat to human health and the economic development of animal husbandry [6]. To date, at least 44 *Cryptosporidium* spp. and more than 120 genotypes have been identified [21]. *Cryptosporidium parvum* and *Cryptosporidium hominis* are the two species that mainly cause cryptosporidiosis in humans, and *C. parvum* shows public health importance with its ability to infect a wide range of host species [22]. For the treatment of cryptosporidiosis in immunocompetent patients, nitazoxanide is the only FDA approved drug; however, it is ineffective in immunocompromized or malnourished individuals [1]. Although the need for a vaccine is acknowledged for human cryptosporidiosis, challenges still exist in terms of deciphering the invasion mechanisms, pathogenicity, and immunogenicity [31].

Lectins are involved various physiological processes, including protein transport, signal transduction, and pathogen identification. They are divided into five categories, namely L-type, C-type, S-type, P-type and Pentraxins based on carbohydrate ligands, biological processes, subcellular localization, and dependence of divalent cations. Their ability to bind carbohydrates includes the following: (a) glucose-mannose, (b) galactose and N-acetyl-D galactosamine, (c) N-acetylglucosamine, (d) L-fucose, and (e) sialic acids specific [28]. The L-type lectin was first discovered in leguminous plants that contain a lectin-leg-like domain [24]. So far, four categories of L-type lectin have been reported: Endoplasmic Reticulum Golgi intermediate compartment-53 (ERGIC-53), ERGIC-53 like protein (ERGL), 36 kDa vesicular integral membrane protein (VIP36), and VIP36-like protein (VIPL). These L-type lectins play a critical role in protein transport and secretion, innate immunity, and the UPR signaling pathway in animals and plants because of their carbohydrate-binding activity [12, 20, 25, 33].

To date, the biological function of the L-type lectin remains unknown in *C. parvum*. In this study, we identify a novel single-pass type I membrane protein, mannose-specific L-type lectin, in *C. parvum* named CpLTL. We show that this protein is a surface membrane protein in the parasite sporozoites, and has binding ability to mannose and the surface of host cells. We also confirm the presence of binding partners of CpLTL in the surface of host cells, thereby providing a molecular basis for subsequent identification of ligands and investigation of the biological role of CpLTL.

## Materials and methods

### Ethical standards

The study protocol was reviewed and approved by the Research Ethics Committee of Henan Agricultural University. Experiments were conducted in accordance with animal ethics guidelines and approved protocols.

### Sequence analyses of CpLTL

CpLTL was identified from the genome sequences of *C. parvum* IOWA in the CryptoDB database (https://www.cryptodb.org/). The functional domain distribution of CpLTL was analyzed by SMART online tools (http://smart.embl-heidelberg.de/) [14]. The amino acid sequences of lectin-leg-like family proteins were identified from the UniProt database and aligned using ClustalW in the MEGA 7 software suite [13].

### Parasite material and *in vitro* cell culture

The oocysts of *C. parvum* (IIdA19G1) have been maintained in the laboratory (International Joint Research Laboratory for Zoonotic Diseases of Henan, Zhengzhou, China) and propagated by infecting neonatal calves. Free sporozoites were prepared by incubating oocysts in PBS containing 0.25% trypsin and 0.75% taurocholic acid (Solarbio, Beijing, China) at 37 °C and 90 rpm for 3 h. Human ileocecal adenocarcinoma (HCT-8) cells (American Type Culture Collection, Manassas, VA, USA) were cultured and maintained in RPMI 1640 medium supplemented with 10% fetal bovine serum, 100 μg/mL penicillin, and 100 μg/mL streptomycin (Solarbio, Beijing, China) at 37 °C in a humidified 5% CO_2_ incubator.

### Cloning, expression and purification of CpLTL

A fragment of the *CpLTL* gene, containing the lectin-leg-like domains (nucleotides 130–852), was amplified via PCR from total genomic DNA isolated from *C. parvum* oocysts using primers 5′–CGGGATCCCATAATGATGACCCTGCATCTGCA-3′ and 5′–GACGTCGACTGCAATAGATACAATGGAAGTCGTAG-3′ (restriction sites are underlined). The PCR amplification was performed under the following conditions: denaturation at 95 °C for 5 min, 35 cycles of amplification at 95 °C for 45 s, 57 °C for 45 s, and 72 °C for 1 min; and a final extension at 72 °C for 10 min. The obtained PCR product was inserted into the pGEX-4T-1 vector (Novagen, Madison, WI, USA). The recombinant plasmid was extracted from DH5α and transfected into *E. coli* Rosetta (DE3) cells (ZOMANBIO, Beijing, China). The induction of CpLTL expression was induced by adding 0.1 mM isopropyl-β-d-thiogalactopyranoside (IPTG) (Solarbio, Beijing, China) at 25 °C and 180 rpm for 3 h. The bacterial solution was broken and centrifuged to obtain the supernatant that was incubated with glutathione sepharose 4B beads (GE Healthcare, Pittsburgh, USA) at 4 °C and 100 rpm for 1 h. The purified protein was examined using sodium dodecyl sulfate-polyacrylamide (SDS-PAGE) on a 10% gel (Solarbio, Beijing, China) and western blotting analysis.

Two 3-week-old female New Zealand white rabbits (SPF) were used to obtain CpLTL-specific polyclonal antibodies, through immunization with purified recombinant proteins as antigens, and pre-immunization serums were collected as the negative control. The specificity of polyclonal antibodies was evaluated using an enzyme-linked immunosorbent assay (ELISA).

### Western blotting analysis of native CpLTL protein

Free sporozoites were prepared as described above, and then suspended in a RIPA lysis buffer (Thermo Fisher Scientific, Carlsbad, CA, USA) and centrifuged at 9148×*g* for 10 min to obtain all parasite protein. 10% SDS-PAGE analysis was performed on the whole parasite protein that was transferred to a polyvinylidene fluoride (PVDF) membrane (Millipore, Billerica, MA, USA). The PVDF membranes were sealed with PBST containing 5% skim milk powder at room temperature for 1 h, and incubated with anti-CpLTL polyclonal antibody and pre-immunization serum. Then, reactive protein bands in the membrane were detected using the Immobilon Crescendo Western HRP substrate (Merck Millipore) and analyzed with an Amersham Imager 680 (GE, Boston, CT, USA).

### Subcellular localization analysis of CpLTL

We examined the localization of CpLTL at different developmental stages of *C. parvum* using an immunofluorescence assay (IFA). Oocysts and free sporozoites were fixed on slides with 4% paraformaldehyde for 30 min to examine CpLTL expression in these stages. Intracellular stages of *C. parvum* were obtained by infecting HCT-8 cells grown on coverslips for 24 and 48 h, fixed with 4% paraformaldehyde and permeabilized with 0.1% Triton X-100 (Solarbio, Beijing, China). The polyclonal antibody and the goat anti-rabbit fluorescent secondary antibody labelled with Alexa Fluor^®^ 594 (Proteintech, Wuhan, China) were diluted with a blocking solution at 1:500. Cell nuclei were counterstained with anti-fluorescence quenching sealing solution (including DAPI) (Solarbio, Beijing, China). Samples were observed and collected images using a BX53 immunofluorescence microscope (Olympus, Tokyo, Japan).

### Expression level analysis of *CpLTL* gene

We examined the expression level of the *CpLTL* gene at different developmental stages of *C. parvum*. Samples were collected at 3, 6, 12, 24, 48 and 72 h post-infection. Total RNA was extracted from wells using TRIzol (Invitrogen, Waltham, MA, USA), and the concentration was measured using a NanoDrop 2000 (Thermo, Waltham, MA, USA). cDNA was synthesized from 2 μg of extracted RNA using a GoScriptTM Reverse Transcription System (Promega, Beijing, China) and analyzed via qPCR. Relative expression levels of the *CpLTL* gene at different time points were homogenized using the expression level of the *C. parvum* 18S rRNA gene. The expression of lactate dehydrogenase (*CpLDH*) was detected in parallel for comparison and quality control. The following primers were used: 5′-GCATCCCAAGTACATAAAGCAA-3′ and 5′-TCTTCCATACAACCAGTCCAAA-3′ for the analysis of the *CpLTL* gene [19], 5′-CTCCACCAACTAAGAACGGCC-3′ and 5′-TAGAGATTGGAGGTTGTTCCT-3′ from the *C. parvum* 18S rRNA gene [11], and 5′-AAGCAAGGTCTTATCACCCAG-3′ and 5′-GCAAAGTAGGCAGTTCCTGTC-3′ from the *CpLDH* gene (cgd7_480) [34]. The relative expression level of the *CpLTL* gene was calculated using a 2^−∆∆CT^ formula as described [5].

### Invasion neutralization assay

The blocking efficiency of anti-CpLTL polyclonal antibodies on the invasion of *C. parvum* was examined using an invasion neutralization assay [4]. Freshly excysted *C. parvum* sporozoites (8 × 10^4^ per well) were suspended in FBS free RPMI-1640 medium containing antibodies or pre-immune serum (1:50, 1:100, and 1:500 dilutions) and added to the plate via medium exchange to allow invasion of host cells at 37 °C for 2 h, with medium alone as controls. After washing with culture medium to remove non-invading sporozoites, and cells were cultured for 24 h. The method for assessment of antibodies against *C. parvum* infection of HCT-8 cells *in vitro* was based on the quantitative real-time reverse transcription-PCR (qRT-PCR) technique [3].

### Evaluation of the binding activity of CpLTL to mannose

CpLTL contains a highly conserved carbohydrate-binding domain, described as mannose binding lectin. Because of the mannose-rich structure on the surface of horseradish peroxidase (HRP), the carbohydrate-binding ability of CpLTL was detected by this feature. GST-CpLTL (GST) proteins were fixed on the 96-well enzyme-labelled plate, washed with PBS and treated with 1% BSA for 1 h, incubated with 100 mMol/L mannose and 50 μg/mL horseradish peroxidase (Sigma-Aldrich, Shanghai, China) in PBS overnight at 4 °C. The positive control group was added to 50 μg/mL horseradish peroxidase (HRP) in PBS, and the negative control group was added to PBS buffer. Then the glycosyl binding activity was evaluated using an ELISA at 405 nm (OD405).

### Analysis of the binding activity of GST-CpLTL to parasites or host cells

The binding activity of GST-CpLTL to parasites was assessed with far-western blotting analysis [26]. The binding activity of GST-CpLTL to host cells was assessed with a protocol similar to ELISA and IFA. For binding to live cells, HCT-8 cells were cultured in 12-well cell culture plates at ~80% confluence and incubated with different dilutions of GST-CpLTL and GST proteins (100, 200, 300, 400, and 500 μM). For binding to fixed cells, HCT-8 cells were fixed with 4% paraformaldehyde, blocked with 1% BSA, and incubated with different dilutions of proteins. Recombinant proteins bound to the HCT-8 cells surface were detected using monoclonal anti-GST antibody, and evaluated using an ELISA at 405 nm (OD405) and an IFA with Alexa Fluor^®^ 594-labelled goat anti-rabbit antibody (Proteintech, Wuhan, China).

### Efficiency of recombinant CpLTL protein and mannose on invasion

The efficiency of GST-CpLTL protein and mannose on invasion of *C. parvum* was examined using qRT-PCR. HCT-8 cells were incubated with different dilutions of GST-CpLTL and GST proteins (100, 200, and 400 μM) at 37 °C for 1 h. Subsequently, oocysts (2×10^6^) were added to HCT-8 cells cultured in a 12-well cell culture plate. Oocysts (2×10^6^) were incubated with different dilutions of mannose (25, 50, 100, and 200 mM) in RPMI 1640 medium containing 10% FBS at 37 °C for 1 h, then added to HCT-8 cells cultured in 12-well cell culture plate. The procedure of the qRT-PCR technique is described above. The method for assessment of recombinant CpLTL protein and mannose against *C. parvum* infection of HCT-8 cells *in vitro* was calculated using the empirical 2^−∆∆CT^ formula [3].

### GST-pull-down assay

Binding partners of CpLTL in the host cells were detected by GST-pull-down assay. HCT-8 cells (2 × 10^7^) were collected by centrifugation at 500×*g*, and extracted according to a Minute™ 146 plasma membrane proteins isolation and cell fractionation kit (Invent Biotechnologies, Ely, UK). GST-CpLTL or GST proteins were bound to glutathione sepharose 4B beads at 4 °C for 2 h, and beads were washed several times using PBS buffer. Then, the beads were incubated with HCT-8 cells plasma membrane proteins in PBS at 4 °C for 3 h, and washed several times using Tween 20 to remove unbound proteins. Each of the samples were examined by SDS-PAGE on a 10% gel followed by silver staining according to quicksilver staining 156 kits (Beyotime, Shanghai, China).

### Identification of LC-MS/MS

The samples obtained from the silver staining of the GST-pull-down assay were sent to Genecreate Biological Engineering (Wuhan. China), and digested with trypsin overnight, then added to the peptide extract (Sigma-Aldrich, Shanghai, China) and desalinated with C18 desalting columns, subjected to liquid chromatography with tandem mass-spectrum (LC-MS/MS) analysis according to standard protocols. The data were retrieved by MaxQuant (V1.6.6.0) software, using the database search algorithm Andromeda [30]. Identified peptides were further mapped to specific proteins by searching the UniProt and CryptoDB databases.

## Results

### CpLTL is a type I transmembrane protein with a signature lectin-leg-like domain

**Figure S1:**
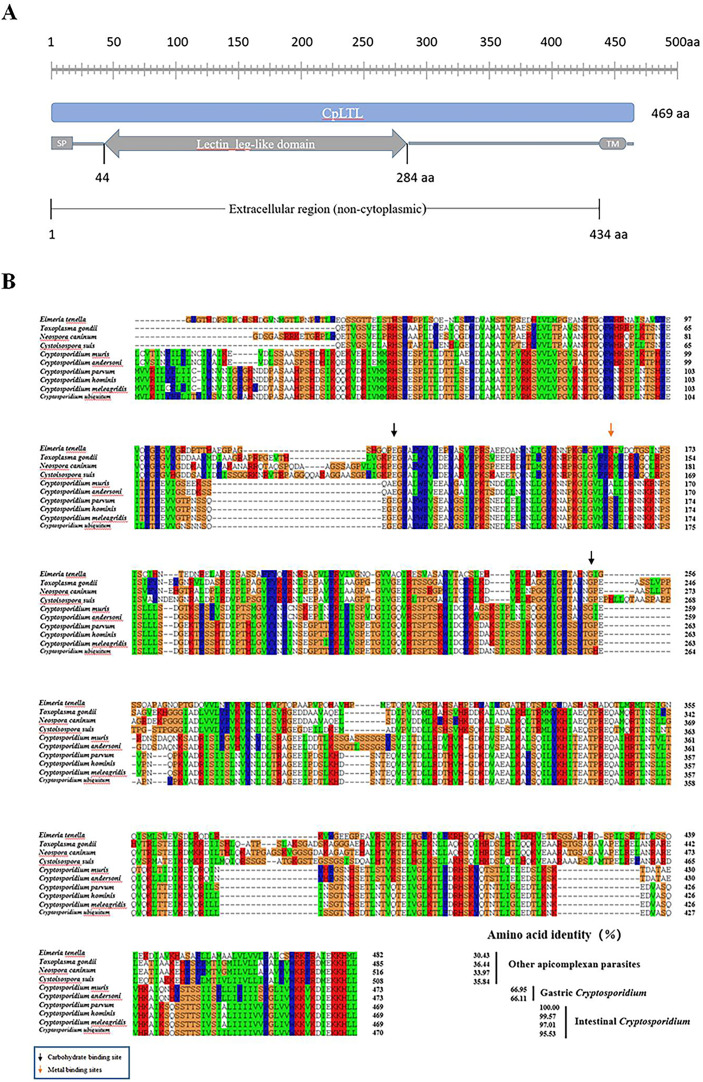
Sequence analyses of CpLTL. (A) Domain organization of CpLTL protein using SMART. SP: signal peptide, TM: transmembrane domain. (B) Amino acid sequences of apicomplexan parasite LTLs were aligned with CpLTL using the Clustal W algorithm. The metal-binding sites were marked with a red arrow, and the carbohydrate-binding sites were marked with a black arrow. Amino acid identities with orthologs from other *Cryptosporidium* species and apicomplexan parasites.

Sequence analysis revealed that CpLTL gene was encoded by a 1364-bp open-reading frame (ORF) predicting 469 amino acids (aa) and was annotated as “ERGIC-53-like mannose binding lectin that is a type I membrane protein, transmembrane domain near C, signal peptide” at CryptoDB (GeneID: cgd6_5140) and GenBank (accession number: XM_627836.1). The SMART online tools predicted that CpLTL is composed of a signature lectin-leg-like domain (44–284 aa), an N-terminal signal peptide (SP) (1~21 aa), and a C-terminal transmembrane domain (TM) (435~457 aa), and it was predicted to be a type I membrane protein with a long extracellular region, belonging to the “legume-like lectin family” (Fig. S1A). Compared to other apicomplexan LTLs, the described carbohydrate-binding sites (E-121 and G-261) were conserved. However, a variation was observed at the metal binding site (S-163) (Fig. S1B). The sequence of CpLTL shared 95.53–99.57% and 66.11–66.95% amino acid identities to the homologs from intestinal (*C. hominis*, *C. ubiquitum*, *C. meleagridis*) and gastric (*C. muris* and *C. andersoni*) *Cryptosporidium* species. CpLTL was also highly divergent from homologs of other apicomplexan parasites, such as those from *Toxoplasma gondii* (36.44% identity), *Neospora caninum* (33.97% identity), *Eimeria tenella* (30.43% identity), *Cystoisospora suis* (35.84% identity), and *Besnoitia besnoiti* (36.80% identity) (Fig. S1B).

### Production of recombinant CpLTL in *Escherichia coli*

**Figure S2:**
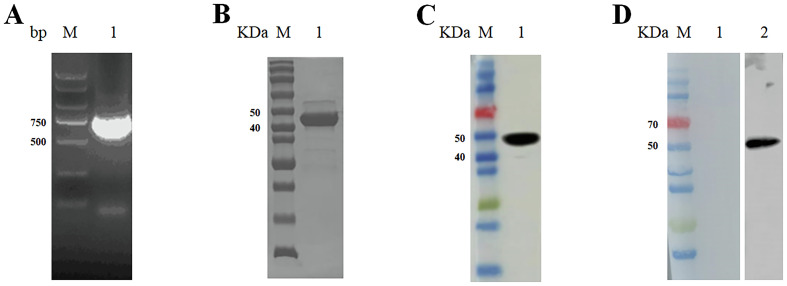
Expression of recombinant CpLTL in *Escherichia coli* and identification of native CpLTL in *C. parvum* sporozoites. (A) PCR amplification of the CpLTL of *C. parvum*. Lane M: 2000 bp DNA ladder maker. Lane 1: CpLTL product. (B) Purity of recombinant CpLTL assessed by SDS-PAGE analysis. Lane M: molecular weight markers, Lane 1: CpLTL purified using Glutathione Sepharose affinity chromatography. (C) Expression of recombinant CpADF in *E. coli* determined via western blotting analysis using an anti-GST tag. Lane M: protein size makers. Lane 1: recombinant CpADF protein. (D) Western blotting analysis of native CpLTL in *C. parvum* sporozoites using pre-immune serum (left panel) and polyclonal antibodies (right panel). Lane M: protein size makers. Lane 1 and lane 2: native protein from sporozoites.

The fragment of *CpLTL* gene was successfully amplified via PCR from the genomic DNA of *C. parvum* using the abovementioned specific primers, and the size was consistent with our expectation (Fig. S2A). The SDS-PAGE analysis revealed that the molecular mass of recombinant CpLTL was similar to the expected sizes of ≈48 kDa (Fig. S2B). Recombinant CpLTL was confirmed via western blotting analysis using anti-GST tag antibodies (Fig. S2C).

### Identification of native CpLTL

To detect native CpLTL protein, polyclonal antibodies were used in western blotting analysis. Polyclonal antibodies against native CpLTL reacted with a protein of ≈52 kDa in lysates of *C. parvum* sporozoites (Fig. S2D), and pre-immune serum did not react with any other proteins (Fig. S2D), which confirmed the specificity of the antibody for CpLTL.

### CpLTL is a surface membrane protein in *C. parvum* sporozoites and expressed during the intracellular development stages

**Figure 1:**
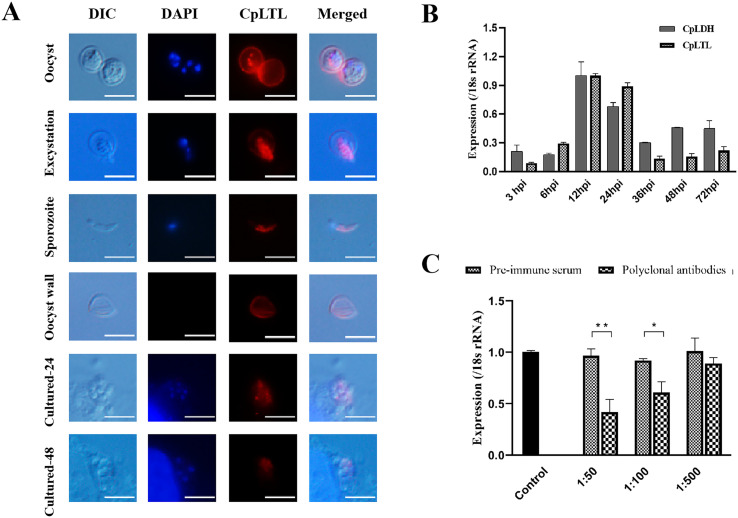
Expression and neutralization of CpLTL in oocysts and developmental stages in *C. parvum*. (A) Expression of CpLTL in oocysts (first panel), excystation (second panel), sporozoites (third panel), oocyst wall (fourth panel), and intracellular developmental stages of *C. parvum* in HCT-8 cell cultures at 24 (fifth panel) and 48 h (sixth panel). The images were taken under differential interference contrast (DIC), with nuclei being counter-stained with 4′, 6-diamidino-2-phenylindole (DAPI), parasites stained by immunofluorescence with Alexa Fluor^®^ 594-labelled secondary antibody, and superimposition of the three images (Merged). Bars = 5 μm. (B) Expression level of the *CpLTL* gene at various *C. parvum* culture times as determined by qRT-PCR. Data from the *Cryptosporidium* 18S rRNA gene were used as an internal control for data normalization. The *CpLDH* gene was detected in parallel as a reference and for quality control. Data are presented as Mean ± SD from three replicate cultivation assays. (C) Neutralization efficiency of polyclonal antibodies against CpLTL in *C. parvum* culture. Oocysts were pre-incubated in medium with 1:500, 1:100, and 1:50 dilutions of pre-immune serum and polyclonal antibodies, with medium alone as a control. Data are presented as Mean ± SD from three replicate assays (*p* < 0.05).

Given that CpLTL was predicted to be a type I membrane protein through sequence analyses, we examined the localization of CpLTL protein during the developmental stages of *C. parvum* using IFA. The results showed that CpLTL was localized to the wall of oocyst and the surface of mid-anterior region of sporozoite (Fig. 1A). At the intracellular development stages (24 and 48 h), polyclonal antibodies against CpLTL reacted with the cytoplasm of merozoites (Fig. 1A). These data suggested that CpLTL may be a surface membrane protein in the parasite sporozoites.

To explore the expression pattern of *CpLTL* gene, we examined the transcript expression of the *CpLTL* gene during the development stages of *C. parvum in vitro* using qRT-PCR. The results showed that the *CpLTL* gene was expressed in *C. parvum* at different developmental stages and had a peak expression at 12 h post-infection (Fig. 1B). Transcript expression of the *CpLTL* gene coincided with the expression profile of CpLTL deposited in the *Cryptosporidium* database (CryptoDB) [16, 18].

### Neutralization efficiency of CpLTL polyclonal antibodies on invasion

The neutralization efficiency of polyclonal antibodies against CpLTL on *C. parvum* invasion was assessed *in vitro*. In comparison with the pre-immune sera-treated culture, anti-CpLTL antibodies partially neutralized invasion of *C. parvum* (the highest inhibition rate = 58.10%, *p* = 0.012) (Fig. 1C). These data showed that CpLTL may play some roles in the invasion of *C. parvum*.

### CpLTL is capable of binding to mannose and the host cell surface

**Figure 2:**
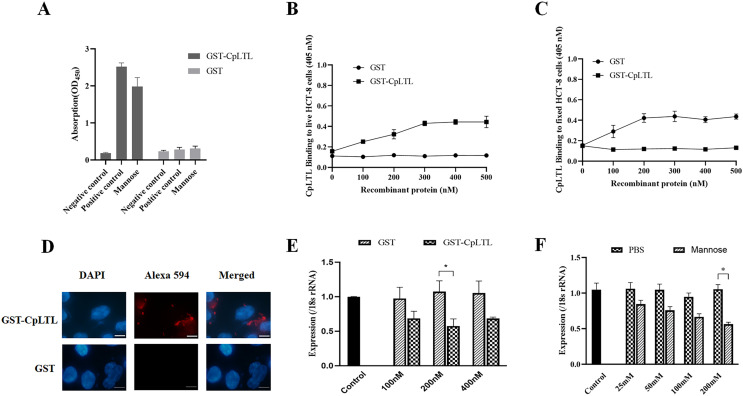
Detection of the binding activity of recombinant CpLTL to mannose and the surface of host cells, and exploration of the efficiency of recombinant CpLTL protein and mannose on invasion. (A) Binding ability of GST-CpLTL to mannose was detected by ELISA. GST was used as an internal control for data normalization. (B, C) The binding activity of GST-CpLTL to the surface of fixed and live host cells was detected by ELISA. GST was used as an internal control for data normalization. (D) The binding activity of GST-CpLTL to the surface of fixed host cells was detected by IFA. Fixed host cells were incubated with GST-CpLTL or GST alone, labelled with anti-GST antibody and Alexa Fluor^®^ 594-labelled secondary antibody. (E) The inhibition efficiency of GST-CpLTL to invasion of *C. parvum* was quantified by qRT-PCR. Host cells were pre-incubated with different dilutions of GST-CpLTL and GST, with medium alone as a control. Data are presented as Mean ± SD from three replicate assays (*p* < 0.05). (F). The inhibition efficiency of mannose to invasion of *C. parvum* was quantified by qRT-PCR. Oocysts were pre-incubated with different dilutions of mannose and PBS, with medium alone as a control. Data are presented as Mean ± SD from three replicate assays (*p* < 0.05).

Because the CpLTL was predicted to be a mannose binding lectin through sequence analyses, we examined the glycosyl binding activity of CpLTL using ELISA. Mannose showed competitive inhibition on the binding of HRP to GST-CpLTL protein, compared with the positive control group (without any monosaccharides). The blank control and GST control group had no binding ability (Fig. 2A). These data indicated that CpLTL may have certain carbohydrate-binding ability with mannose.

We explored whether the carbohydrate-binding ability of CpLTL occurred in the parasite or host cells. In far-western blotting analysis, GST-CpLTL was used to probe the lysates of *C. parvum* sporozoites, and it was found that GST-CpLTL and GST proteins did not react with any proteins (data not shown). By contrast, the binding ability of GST-CpLTL on the surface of fixed and live host cells occurred in a dose-dependent and saturable manner, but not on host cells incubated with GST protein (Figs. 2B and 2C), and these findings were further confirmed by IFA. Bright fluorescent signals were detected on the surface of fixed host cells incubated with GST-CpLTL protein using an anti-GST antibody, but not on cells incubated with GST protein (Fig. 2D). These data implied that CpLTL may be a potential host cell surface binding protein.

### Inhibition efficiency of recombinant CpLTL and mannose on invasion

The abovementioned experiments illustrated that the binding of CpLTL to host cells might primarily occur through the interaction between CpLTL and oligosaccharide chains of glycoproteins on the host cell surface. To determine the function of CpLTL during the invasion of *C. parvum*, we conducted competition inhibition tests for both the recombinant CpLTL and mannose. Preincubation of host cells with GST-CpLTL protein resulted in statistically significant inhibition of invasion compared with GST protein. It was found that GST-CpLTL had the most significant inhibition effect on the invasion of *C. parvum* at 200 nM (inhibition rate = 42.5%, *p* = 0.043) (Fig. 2E). Preincubation of oocysts with mannose partially blocked the invasion of *C. parvum* sporozoites into host cell compared with negative control group (PBS) (the highest inhibition rate = 43.8%, *p* = 0.031) (Fig. 2F).

### Host cells contain potential binding partners for CpLTL

**Figure S3:**
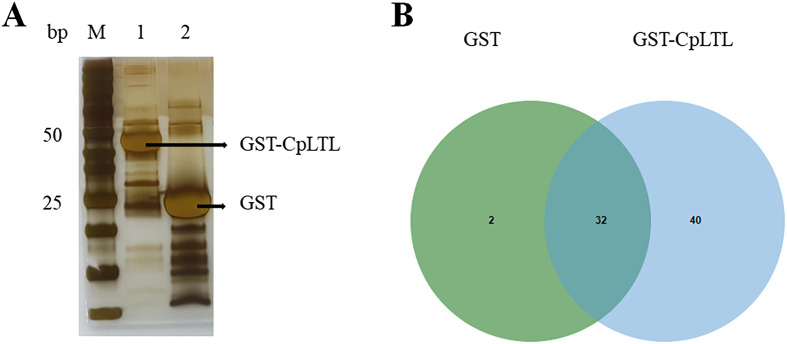
Identification of host proteins interacting with CpLTL by GST-pull down combined with LC-MS/MS. (A) Silver-stained one-dimensional SDS-PAGE analysis of GST-CpLTL. Line M: marker. Line 1: GST-CpLTL. Line 2: GST control. Line 3: PBS control. (B) The Venn diagram of the differentiated proteins was obtained by LC-MS/MS identification.

To identify host cell proteins that interacted with CpLTL, HCT-8 cell plasma membrane proteins were extracted and incubated with the GST-CpLTL and GST proteins, and analyzed on an SDS-PAGE gel by silver staining (Fig. S3A). Proteins that were detected bound to the GST-CpLTL were largely different from proteins bound to the GST control. A total of 72 proteins were identified from the GST-CpLTL sample by LC-MS/MS analysis, in which 32 proteins were also identified from the GST sample (Fig. S3B), resulting in 40 effective proteins obtained in the GST-CpLTL sample (Table S1). By analyzing the subcellular localization, glycosylation sites, and biological functions of these 40 effective proteins when the confidence conf ≥ 95% and unique peptides ≥ 2, two host cells proteins (immunoglobulin heavy constant alpha 1 (accession: P01876) and metabotropic glutamate receptor 1 (accession: Q13255)) showed high matches and therefore should be prioritized for further investigation.

## Discussion

Owing to the carbohydrate binding specificity of lectins, they mediate parasite-host cell interactions and play some important roles in parasites adhesion and invasion [27, 29]. So far, some functional lectins have been reported in parasites, such as a Gal/GalNAc specific lectin. P30 which was isolated from *C. parvum* and *Cryptosporidium hominis* sporozoites by Gal-affinity chromatography, localized in the mid-anterior region of sporozoites. P30 could combine with gp900 and gp40 (mucin-like glycoprotein) to mediate infection and might serve as a potential target to intervene in the invasion of *C. parvum* [2]. A novel C-type lectin, CpClec, was localized in the mid-anterior region of sporozoites, which mediated *C. parvum* attachment and infection by binding to sulfated proteoglycans on intestinal epithelial cells in a Ca^2+^-dependent manner [17]. A galectin-like micronemal protein 1 (TgMIC1) from *Toxoplasma gondii* could specifically bind sialylated oligosaccharides to assist in parasite adhesion and participate in the folding and assembly of MIC1/4/6 complexes [23]. These interactions could be exploited to develop novel therapeutics, targeting the adhesion and invasion, and thus be helpful in eradicating the widespread parasite diseases.

As a type I membrane protein on the parasite cell surface, CpLTL may serve as an adhesive molecule for sporozoites during their moving and gliding on the gastrointestinal epithelial surfaces, penetration of mucosal layers, and attachment/invasion of host cells at the infection sites. CpLTL was localized to the wall of oocysts, illustrating the potential usage of CpLTL as a marker for *C. parvum* detection *in vitro*. Also, *C. parvum* could actively invade host cells using the action of secretory organelles, such as micronemes, rhoptries, and dense granules, which stored many proteins related to adhesion and invasion, such as GP900, CpROM1, ROP1, and MEDLE-1 [8–10, 15]. Similar to these proteins, CpLTL was also localized in the mid-anterior region of sporozoite. It is speculated that CpLTL may be secreted from the apical secretory organelles during invasion. Invasion neutralization assay suggested that CpLTL as a surface protein likely plays a role during the invasion of sporozoites into host cells.

Another open question is the identity of potential CpLTL-binding partners observed by GST-pull-down. Proteomic analysis identified 40 effective host cell proteins from the areas corresponding to some bands recognized by CpLTL in the GST-pull-down assay. Because CpLTL is a single-pass type I membrane protein containing a highly conserved carbohydrate-binding domain, it may interact with glycoproteins on the surface of host cells membrane to mediate the adhesion and invasion of *C. parvum.* Therefore, by analysing the subcellular localization, glycosylation sites, and biological functions of these 40 effective proteins when the confidence conf ≥95% and unique peptides ≥2, two proteins (Immunoglobulin heavy constant alpha 1 and metabotropic glutamate receptor 1) showed high matches and should be considered potential candidate binding partners for further investigation. The first protein was annotated as “Immunoglobulin heavy constant alpha 1” in UniProt. It was located in the cell membrane and had 11 glycosylation sites that could serve as receptors and bind to specific antigens, triggering the clonal expansion and differentiation of B lymphocytes into immunoglobulins-secreting plasma cells [32]. The other was annotated as “Metabotropic glutamate receptor 1” in UniProt. It was located in the cell membrane and had 4 glycosylation sites, which could participate in multiple signaling pathways, cell proliferation, and differentiation in the body [7]. Nonetheless, the exact roles played by CpLTL remain an intriguing question that is worth further investigation.

## Conclusions

In this study, we characterized the CpLTL as a type I transmembrane protein with a signature lectin-leg-like domain. Analysis of subcellular localizations indicated the surface presentation of CpLTL in sporozoites of *C. parvum*. Through antibody neutralization and competitive inhibition assays, it was demonstrated that CpLTL participated in the invasion of *C. parvum*, potentially in a manner of interacting with host cell membrane proteins. The identification of two host cell membrane proteins supported the interaction between CpLTC and host cells. Future clarification of interactions between CpLTL and the identified host cell proteins will be necessary to understand the function of CpLTL during the adhesion and invasion of *C. parvum*.

## Supplementary material

The supplementary material for this article can be found at https://www.parasite-journal.org/10.1051/parasite/2024051/olm.

*Table S1*: List of proteins identified by LC-MS/MS analysis from the bands recognized by CpLTL in GST-pull down assay.
