# Supplementary material for: A single-pass type I membrane protein, mannose-specific L-type lectin, potentially involved in the adhesion and invasion of Cryptosporidium parvum
Source: Parasite. 2024 Aug 29;31:51. doi: 10.1051/parasite/2024051 (PMC11363900; doi:10.1051/parasite/2024051)
Supplement: Supplementary file 1 — Table S1: List of proteins identified by LC-MS/MS analysis from the bands recognized by CpLTL in GST-pull down assay. [file parasite-31-51-s1.pdf]

Table 1 List of proteins identified by LC-MS/MS analysis from the bands recognized by CpLTL

Name	Length	Location	Glycosylation(number)	Uniprot
**Metabotropic glutamate receptor 1**	**1194**	**Cell membrane**	**4**	**Q13255**
**Immunoglobulin heavy constant alpha 1**	**353**	**Cell membrane**	**11**	**P01876**
Heat shock protein beta-1	205	Crtoplasm、Nucleus	-	P04792
Corneodesmosin	529	Secreted	1	Q15517
GTPase 2	406	Mitochondrion	-	Q9H4K7
Interleukin-36 gamma	169	Crtoplasm、Secreted	-	Q9NZH8
Protein S100-A7	101	Crtoplasm、Secreted	-	P31151
Plakophilin-1	747	Nucleus	-	Q13835
Zinc-alpha-2-glycoprotein	298	Secreted	2	P25311
Caspase-14		Crtoplasm、Nucleus	-	P31944
Gamma-glutamylcyclotransferase	353			O75223
	188	Cytosol	-	
Protein-glutamine gamma-glutamyltransferase K	817	Cytosol、Cell membrane	-	P22735
Ribonuclease 7	156	Secreted	1	Q9H1E1
Lactotransferrin	710	Cytoplasmic granule	4	P02788
Protein disulfide-isomerase A5	519	Endoplasmic reticulum	-	Q14554
Extracellular matrix protein 1	540	Secreted	3	Q16610
Cystatin-S	141	Secreted	-	P01036
Rho GTPase-activating protein 24	748	Cytoplasm、Cytoskeleton	-	Q8N264
Proteasome subunit beta type-6	239	Cytoplasm、Nucleus	-	P28072
Pyruvate kinase PKM	531	Cytoplasm、Nucleus	-	P14618
Eukaryotic translation initiation factor 6	245	Cytoplasm、Nucleus	-	P56537
Gasdermin-A	445	Cytoplasm	-	Q96QA5
Carboxypeptidase A4	421	Secreted	1	Q9UI42
Proteasome subunit beta type-4	264	Cytoplasm、Nucleus	-	P28070

Heat shock cognate 71 kDa protein	646	Cell membrane	-	P11142
Gamma-glutamyl hydrolase	318	Secreted	4	Q92820
Histidine ammonia-lyase	657	Cytosol	-	P42357
EGF-like repeat and discoidin I-like	480	Secreted	3	O43854
Proteasome subunit alpha type-6	246	Cytoplasm、nucleus	1	P60900
Proteasome subunit alpha type-1	263	Cytoplasm、nucleus	1	P25786
Elongation factor 2	858	Cytoplasm、nucleus	-	P13639
Proteasome subunit alpha type-7	248	Cytoplasm、nucleus	1	O14818
Arachidonate 12-lipoxygenase	701	Cytoplasm	-	O75342
Serpin B7	380	Cytoplasm	-	O75635
Alpha-2-macroglobulin-like protein 1	1454	Secreted	5	A8K2U0
Protein TESPA1	521	Cytoplasm	-	A2RU30
Lysine-specific demethylase 4B	1096	Nucleus	-	O94953
Max-interacting protein 1	228	Nucleus	-	P50539
Calmodulin-like protein 5	146	Secreted	-	Q9NZT1
WD repeat and FYVE domain-containing protein 3	3526	Cell membrane	-	Q8IZQ1
